# Utilization of a nonionic surfactant for improved corrosion resistance of carbon steel in simulated fuel-grade ethanol

**DOI:** 10.1039/c8ra02936a

**Published:** 2018-06-07

**Authors:** M. A. Deyab

**Affiliations:** Egyptian Petroleum Research Institute (EPRI) PO Box 11727, Nasr City Cairo Egypt hamadadeiab@yahoo.com + 20222747433 +201006137150

## Abstract

In this study, a nonionic surfactant (PEG-40 hydrogenated castor oil, Abbrev. PEG-40 HCO) was used to improve the corrosion resistance of carbon steel in simulated fuel-grade ethanol (SFGE). The studies were conducted using cyclic voltammetry (CV) and potentiodynamic polarization techniques and complemented by scanning electron microscopy (SEM) investigations. The presence of water and chloride ions in SFGE strongly influences the electrochemical behavior of carbon steel. Polarization curves indicate that PEG-40 HCO has good inhibition efficiency and behaves as a mixed inhibitor. The inhibition efficiency increases with the concentration of PEG-40 HCO within the range of 20 to 100 ppm, reaching a maximum value of 93.8%. The adsorption of PEG-40 HCO obeys the Langmuir adsorption isotherm. Quantum chemical calculations were evaluated to confirm experimental results.

## Abstract 2

In this study, a nonionic surfactant (PEG-40 hydrogenated castor oil, Abbrev. PEG-40 HCO) was used to improve the corrosion resistance of carbon steel in simulated fuel-grade ethanol (SFGE).

## Graphical abstract

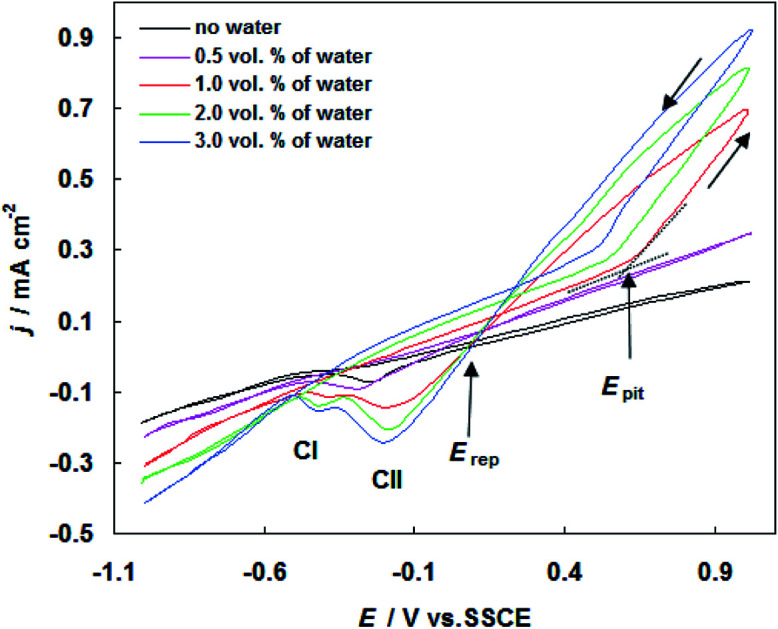

## 1. Introduction

The increasing need for alternative energy has propelled the search for new resources, and one of these is ethanol.^1^ With the increasing demand for ethanol, fast, cheap and safe transportation by pipelines is needed to satisfy the demand. However, pipeline transport of ethanol poses potential problems due to corrosion-related damage.^2–4^ Previous studies of metallic materials in methanol and ethanol show that significant pitting corrosion can be initiated in these alcoholic solvents.^5,6^ These corrosion failures can strongly cause degradation of in-service equipment, in either the pipeline transportation industry or fuel manufacturing industry. The corrosiveness of the fuel ethanol depends on the content and types of contaminants. Water and chloride ions are expected to be present as contaminants in small amounts in commercial fuel ethanol and may affect the corrosion behavior of the materials it comes into contact with.^7^

Prevention of the corrosion in ethanol is possible by creating a barrier between the steel surface and the ethanol. The selection of the proper inhibitor must be done carefully because the selected inhibitor may emulsify and/or foam. The inhibitor should have adequate properties to adsorb to the steel surface to form a strong barrier film.^8–10^

In this study, nonionic surfactant (PEG-40 HCO) was tested as cheap, non-toxic and environmentally acceptable inhibitor for carbon steel corrosion in SFGE. PEG-40 HCO is a non-ionic surfactant which enables oils to be solubilized into the water.

In order to achieve these objectives, cyclic voltammetry and potentiodynamic polarization methods besides quantum chemical calculations were carried out in this study. Some SEM examinations of the electrode surface have been carried out.

## 2. Experimental details

Specimen of carbon steel with chemical composition (wt%), 0.06 C; 0.06 Si; 0.7 Mn; 0.005 P; 0.001 S; 0.012 Ni; 0.015 Cr; 0.004 Mo; 0.002 V; 0.02 Cu and 99.12 Fe, were machined to obtain cylindrical electrode, which it was sealed in epoxy resin, with a circular cross-section area (0.45 cm^2^) exposed to the electrolyte. Before each experiment, this electrode was mechanically polished with emery paper up to 600 grit, rinsed with distilled water, alcohol and acetone and dried.

The chemical composition of the simulated fuel-grade ethanol SFGE based on ASTM D-4806 used in this study is composed of 98.5 vol% of ethanol, 0.5 vol% methanol, 1.0 vol% of water, 56 ppm of acetic acid and 32 ppm NaCl.^11^ The physicochemical characteristics of the ethanol SFGE are listed in Table 1.

**Table 1:** The physicochemical characteristics of the ethanol SFGE

Property	Unit	SFGE
Appearance	—	Colorless liquid
Odor	—	Alcoholic odor
Physical state	—	Liquid
Boiling point	°C	77
Viscosity at 25 °C	mPa s	1.089
Density at 20 °C	g cm^−3^	0.828
Solubility in water	—	Miscible
Refractive index	—	1.3823
Water content	wt%	1.0
Methanol content	wt%	0.5
Acetic acid content	ppm	56
NaCl content	ppm	32
pH	—	5.7

The nonionic surfactant, namely PEG-40 hydrogenated castor oil PEG-40 HCO C_57_H_110_O_9_(CH_2_CH_2_O)_*n*_ where *n* = 40, was obtained from Cationa Chemical Corporation Company.

CV and potentiodynamic polarization studies were carried out using a potentioscan type (Potentiostat/Galvanostat EG&G model 273) connected with a personal computer. A platinum foil was used as the auxiliary electrode, Ag/AgCl electrode (SSCE) in which the outer compartment is filled with 1.0 M LiCl in ethanol was used as the reference electrode. The reference electrode (SSCE) separated from the test solution by Vycor junction (frit) that serves as a salt bridge. The liquid junction potential was not taken in account since that the experiments were performed comparatively in absence and in the presence of the corrosion inhibitor. All the experiments were carried out at a scan rate 1.0 mV s^−1^. The CV (*E*–*j)* curves were recorded by sweeping linearly the potential from the starting potential (−1.0 V) into the positive direction till a required potential value and then reversed with the same scan rate till the starting potential to form one complete cycle. Potentiodynamic polarization curves were obtained by changing the electrode potential automatically from −250 to +250 mV *versus* open circuit potentials. The logarithmic current density was plotted against the electrode potential. These polarization curves exhibit Tafel-type behavior.

The morphology of the carbon steel surface of some samples was determined after the desired electrochemical tests by employing scanning electron microscopy (SEM). SEM was performed using a JEOL/Quantek detector.

All solutions were prepared from analytical grade chemical reagents using doubly distilled water and were used without further purification.

The solutions temperatures were adjusted to within ±0.2 °C using a water thermostat.

Quantum chemical calculations were performed depends on the based density function theory (DFT) in Materials Studio 6.0 using VAMP module and Accelrys software. The Quantum parameters such as the energy of the highest occupied molecular orbital (*E*_HOMO_), the energy of the lowest unoccupied molecular orbital (*E*_LUMO_), energy gap (Δ*E*) between LUMO and HOMO and were calculated.

## 3. Results and discussion

### 3.1. Electrochemical profile of carbon steel in SFGE and effect of water

**Fig. 1:**
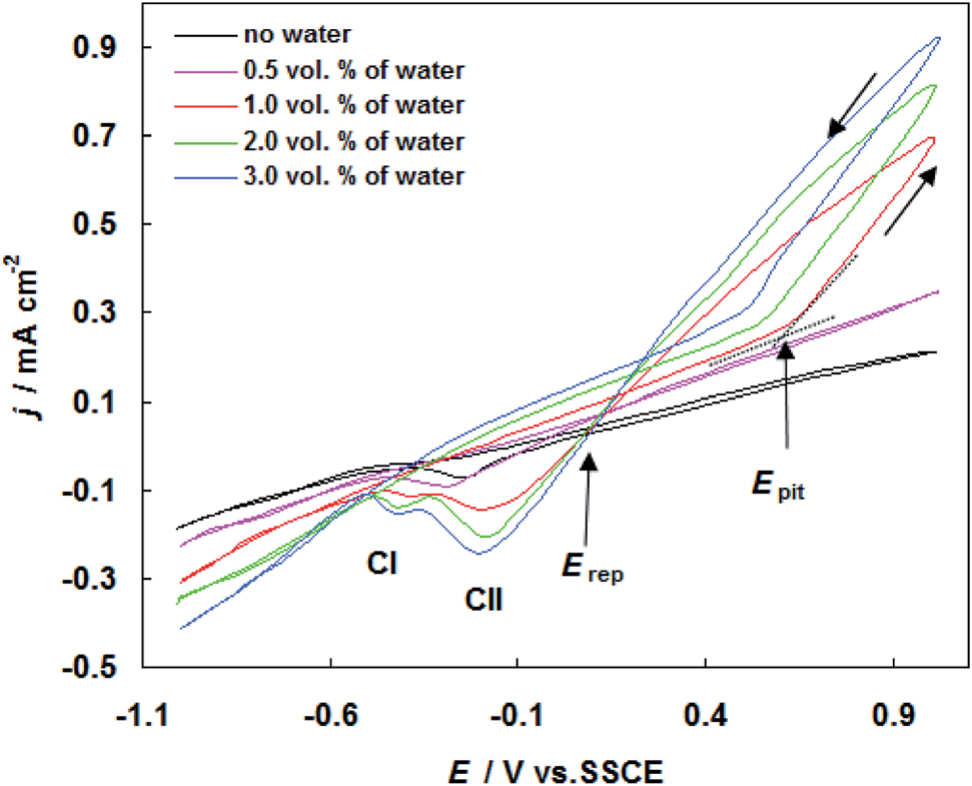
Cyclic voltammograms for carbon steel in SFGE in the absence and presence of different concentration of water at 298 K.

In order to ascertain the electrochemical profile of carbon steel in SFGE, CV experiments were performed to characterize the reduction and oxidation processes on the metal surface. Fig. 1 shows the cyclic voltammogram of carbon steel in SFGE in the absence and presence of different concentration of water. The cyclic voltammetric scan started at −1.0 V *versus* SCE and was swept first in the anodic direction. Inspection of the data reveals that, on positive going sweep, the cathodic current decreases continually and changes its sign at corrosion potential (*E*_corr_).

In the absence of water the electrooxidation of the metal was visible from −0.10 V to 1.0 V *versus* SCE. The addition of water to SFGE led to the shifting of the electrodissolution of the metal to more negative values (*i.e.* −0.162 V, −0.260 V, −0.306 V and −0.355 V *versus* SCE).

Data in Fig. 1 shows that the carbon steel in alcoholic solvent, usually exhibits a poor passivity.^12^ This means that anodic polarization curves display active electrochemical behavior without the formation of an oxide protective film (passive layer) on the metal surface.

When the curve sweeping back in the reverse direction, the cathodic sweep exhibits one cathodic peak (CI), which are probably related to the reduction of hydrogen and iron oxides. It clear form Fig. 1 that the anodic current density values increased in the presence of water. No distinguishable pits are found on the carbon steel surface exposed to the SFGE with less than 0.5 vol% water. As the water content increases from 1.0 to 3.0 vol%, the cyclic voltammograms of carbon steel in SFGE remarkable changes. It is observed that at a certain critical potential (pitting potential *E*_pit_), the anodic current density increases steeply without any sign for oxygen evolution. The rapid rise in anodic current density at *E*_pit_ indicates initiation and growth of pitting attack.^13^

At the reversing the potential sweep, the reverse (pitting) current exhibits a positive hysteresis loop. This represents the property of pitting corrosion. This indicates also that pitting process continues even after scan reversal, because of the autocatalytic character of pitting. The presence of hysteresis loop in CV curve elucidates a retard in repassivation process when the potential is reversed toward the negative direction. Upon reversing scan, the pitting current reaches to zero values at a certain potential known as the repassivation potential (*E*_rep_).^14^ In this stage, all pits become repassivate.^14^ The repassivation process could be achieved by removal of accumulated Cl^−^ ions from the pits by diffusion.^14^

At the reversing scan, the potential is scanned negatively to cause the reduction processes. The corresponding peaks CI and CII are due to the reduction of corrosion product and pitting corrosion products precipitate on the electrode surface, respectively.

It is clear also that the values of *E*_pit_ move in the active direction, with increase in water concentration from 1.0 to 3.0 vol%. Effect of water in non-aqueous solvents, such as ethanol, has been widely studied. Some researchers have concluded that the greater solubility of corrosion product and increase of the hydrated proton concentration in presence of water result in aggressive general and pitting corrosion.^15,16^ The influence of water on pitting corrosion susceptibility of carbon steel in SFGE is mainly due to the ethanol/water solvation and the balance between the surface passivation reactions and the passivity breakdown in such system.

**Fig. 2:**
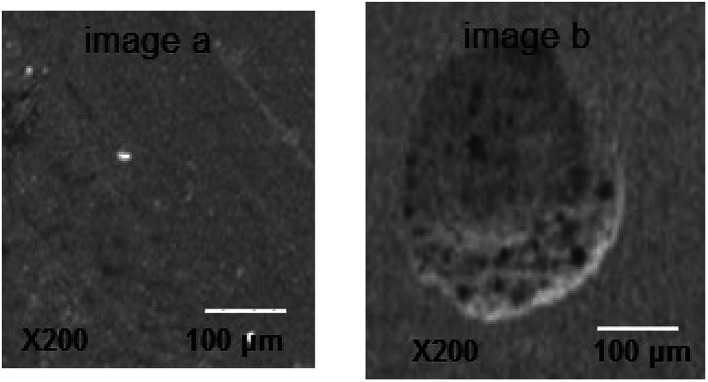
SEM micrographs of the electrode surface in SFGE containing 3.0 vol% water before (image a) and after (image b) *E*_pit_.

The scanning electron microscope was used to characterize the carbon steel surface after the CV test in SFGE before and beyond *E*_pit_. No distinguishable pits are observed on the metal surface exposed to the SFGE before *E*_pit_ under the scanning electron microscope (see image a in Fig. 2). Beyond *E*_pit_ SEM observations reveal the presence of pits on the metal surface after removing the corrosion products (see image b in Fig. 2). These observations comply with electrochemical data, where the oxide layer beyond *E*_pit_ becomes very weak and this leads to the initiation of pitting corrosion on the metal surface. In this case, the breakdown of oxide film is due to the adsorption of Cl^−^ ions on the oxide film, forming an electric field between the oxide/SFGE interfaces. At *E*_pit_ value, the adsorbed Cl^−^ ions penetrate the oxide film and form pits.

**Fig. 3:**
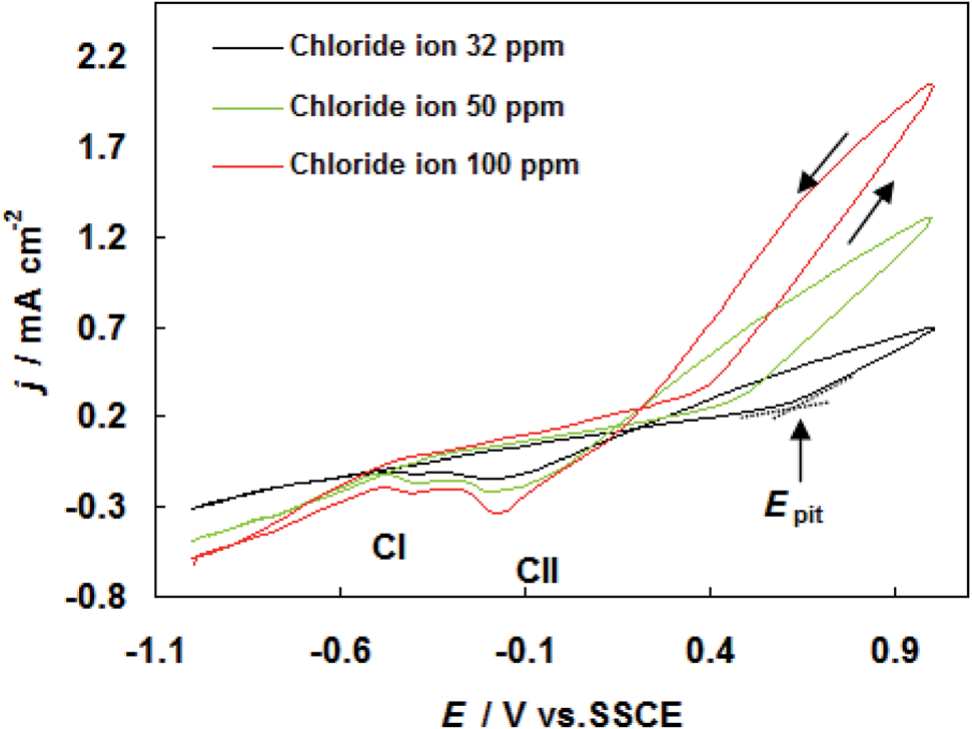
Cyclic voltammograms for carbon steel in SFGE containing 1.0 vol% water in the presence of different concentrations of chloride ions at 298 K.

**Fig. 4:**
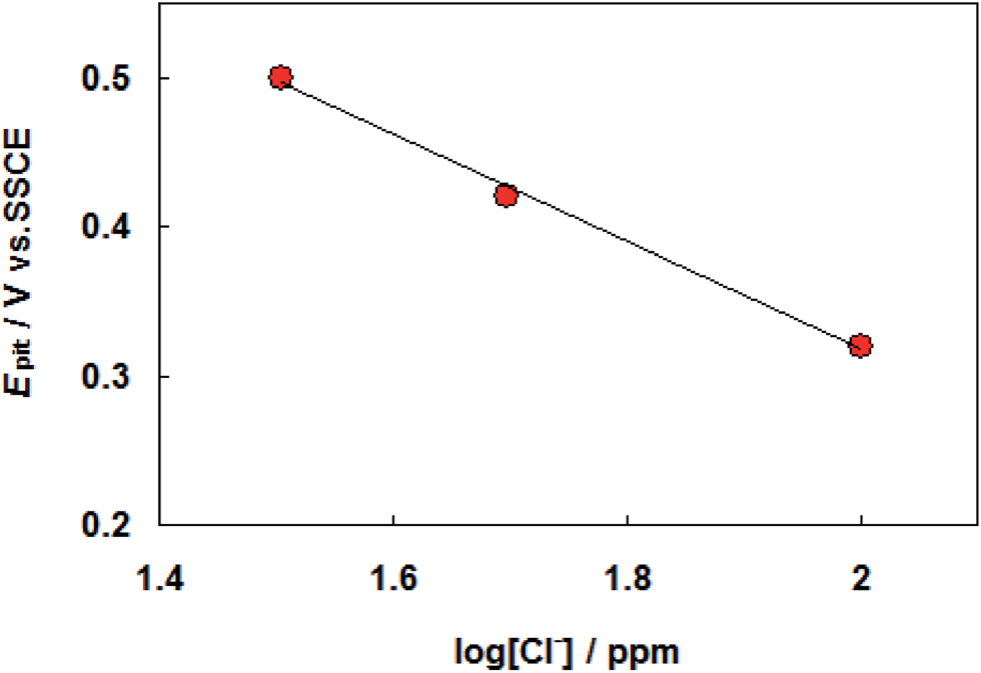
Relation between *E*_pit_*versus* log[Cl^−^] for carbon steel in SFGE.

The results of cyclic voltammograms of carbon steel in SFGE containing 1.0 vol% water in the presence of different concentrations of chloride ions are shown in Fig. 3. The data of Fig. 3 clearly show that, the anodic current density value increases with increasing Cl^−^ ion concentration, indicating the aggressiveness of Cl^−^ ion toward the corrosion process of carbon steel. This behavior could be attributed to the formation of the soluble complex between Fe^2+^ and Cl^−^ ion.^17^ Such complexing processes lead to a further decrease in the free Fe^2+^ ion concentration at the electrode surface. The Cl^−^ ions can be adsorbed on the bare metal surface in competition with OH^−^ ions. As a result of high polarizability of the Cl^−^ ions, the Cl^−^ ions may adsorb preferentially.^18^ The adsorbed Cl^−^ ions can penetrate through the metal surface layer especially at its point defects and flaws and initiate pitting.^18^ Moreover an increase in Cl^−^ ions concentration shifts the pitting potentials toward a more negative (active) direction corresponding to decrease the resistance to pitting. The dependence of the pitting potential on the concentration of chloride ions is given in Fig. 4 which shows *E*_pit_*vs.* log[Cl^−^] whereby a straight line obtained according to the following equation:

1 *E*_pit_ = *a* − *b* log[Cl^−^]

where *a* and *b* are constants depending on the metal composition, electrolyte composition, *etc.* From eqn (1) it is possible to estimate the maximum chloride level above which pitting is expected to occur immediately in relevant environment.

It is obvious to observe from Fig. 3 that an increase in Cl^−^ ion concentration increases the current density of the two cathodic peaks (CI and CII).

### 3.2. The effect of PEG-40 HCO

**Fig. 5:**
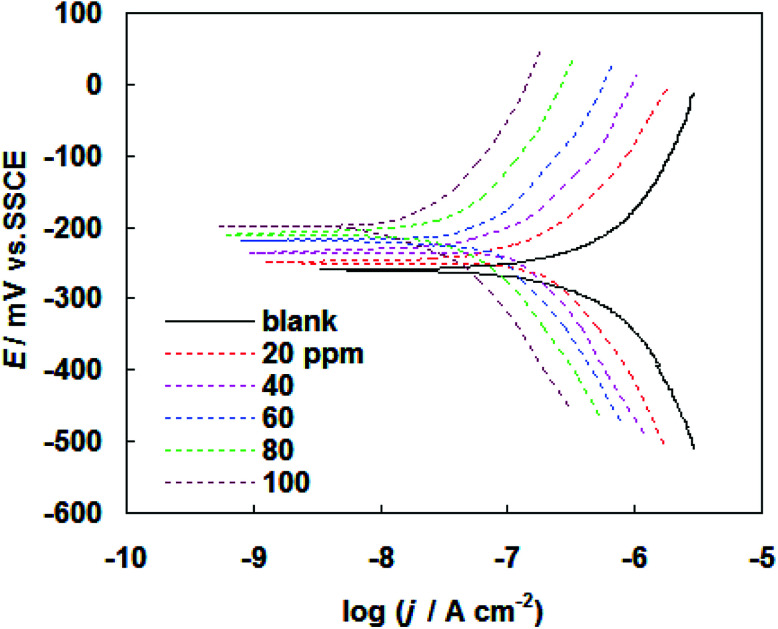
Tafel polarization curves for carbon steel at 298 K in SFGE in the absence and presence of different concentration of PEG-40 HCO.

The corrosion behavior of carbon steel in SFGE in the absence and presence of PEG-40 HCO was investigated by potentiodynamic polarization method. Tafel polarization curves for carbon steel at 298 K in SFGE in the absence and presence of different concentration of PEG-40 HCO are given in Fig. 5. Table 2 shows the electrochemical corrosion kinetic parameters, *i.e.* corrosion potential (*E*_corr_) and corrosion current density (*j*_corr_) obtained from the Tafel extrapolation of the polarization curves. Table 2 also included percentage inhibition efficiency (*η*_j_%). The inhibition efficiency *η*_j_% was obtained from the following equation:^19^2
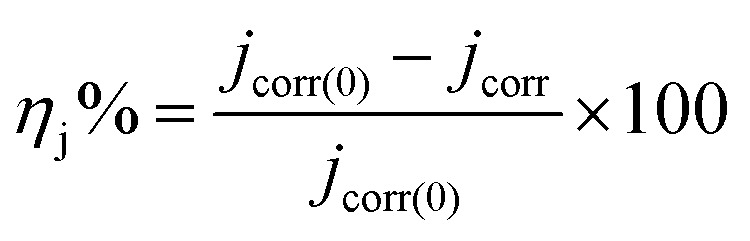
where *j*_corr(0)_ and *j*_corr_ are the uninhibited and inhibited corrosion current densities, respectively. These results show that the inhibition efficiency increased, while the corrosion current density decreased with the addition of PEG-40 HCO. The data clearly show that the addition of PEG-40 HCO shift *E*_corr_ to more positive values. An inhibitor can be classified as cathodic or anodic if the difference in corrosion potential is more than 85 mV with respect to the *E*_corr_ of the blank.^20^ Such results will indicate that the PEG-40 HCO act as a mixed-type inhibitor with predominant anodic effectiveness. These results show that the PEG-40 HCO can retard both anodic and cathodic reactions.^21^ The inhibition efficiency afforded by PEG-40 HCO may be attributed to the adsorption of this compound at the metal/corrosive solution interface. The adsorption process takes place *via* ion pair and ion exchange mechanism by its ethylene oxide groups while its hydrophobic chain are oriented towards the corrosive medium.^22^

**Table 2:** Electrochemical parameters and the corresponding inhibition efficiency of PEG-40 HCO for carbon steel in SFGE in the absence and presence of different concentration of PEG-40 HCO at 298 K

PEG-40 HCO, ppm	*E*_corr_, mV (SSCE)	*j*_corr_, nA cm^−2^	*η*_j_%
Blank	−260	630	—
20	−248	263	58.2
40	−235	190	69.8
60	−219	141	77.5
80	−212	96	84.7
100	−198	39	93.8

The adsorbed PEG-40 HCO molecules on the metal surface may form a surface film, which acts as a physical barrier to restrict diffusion of ions to or from the metal surface and hence retard the corrosion process. The interactions of the adsorbed PEG-40 HCO molecules with surface metal atoms may prevent the metal atoms from participating the anodic reaction of corrosion. This simple blocking effect decreases the number of surface metal atoms participating and hence decreases corrosion. It has been observed that the inhibition efficiency increased with increase in surface coverage by inhibitor molecules. The high inhibition efficiency is due to the bonding of adsorbed PEG-40 HCO molecules on to the metals. The strong bonding is mainly attributed to higher number of ethylene oxide group, present in the adsorbate molecules.^23^

### 3.3. Adsorption considerations

Basic information on the interaction between the PEG-40 HCO molecules and carbon steel surface in SFGE can be provided using the adsorption isotherm. For this purpose, the values of surface coverage (*θ* = *η*_j_%/100) at different concentrations of PEG-40 HCO in SFGE at 298 K were calculated to explain the best isotherm to determine the adsorption process from the experimental data obtained. Attempts were made to fit these *θ* values to various isotherm including Frumkin, Langmuir, Temkin, Freundlich isotherms. By far the best fit is obtained with the Langmuir isotherm. Langmuir adsorption isotherm is described by the following equation:^24^3
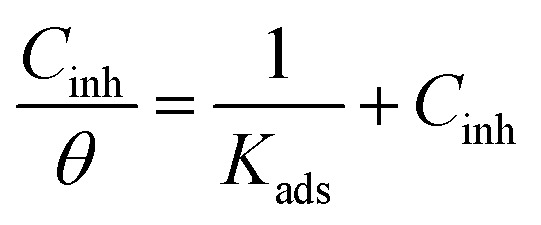
where *K*_ads_ is the equilibrium constant of the adsorption reaction, *C*_inh_ is the inhibitor concentration in the bulk of the solution.

**Fig. 6:**
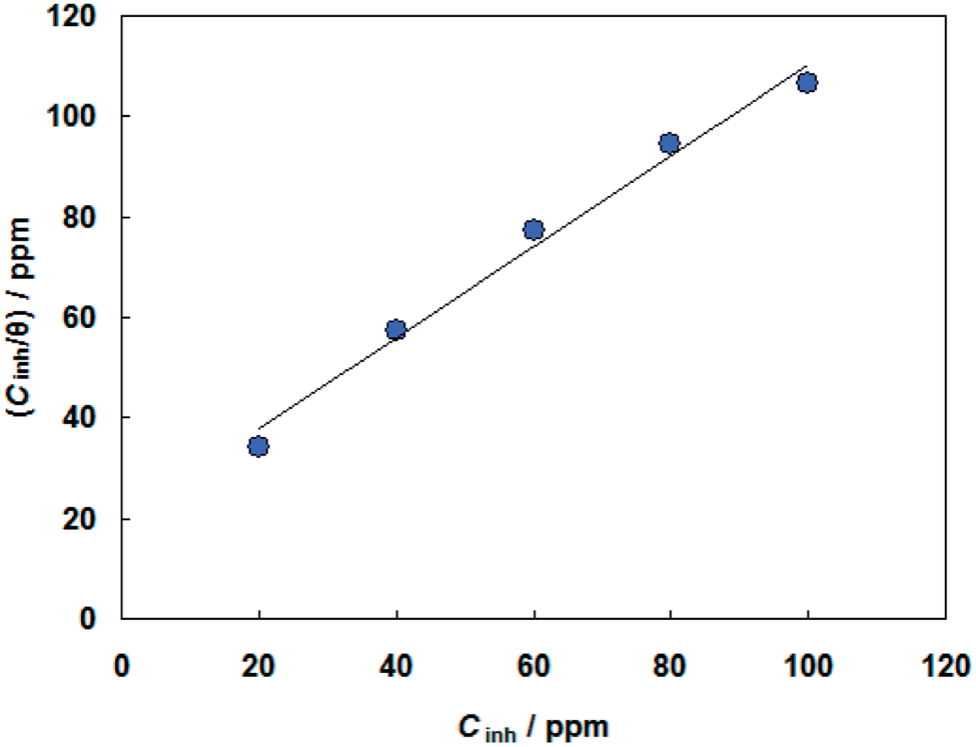
Langmuir's adsorption isotherm plot for the adsorption of PEG-40 HCO in SFGE.

Fig. 6 shows the relationship between (*C*_inh_/*θ*) and *C*_inh_. The obtained plot shows that the linear correlation coefficients (*R*^2^ = 0.9867) are almost equal to unity and the slope of line are very close to unity (slope = 0.992), which indicates that the adsorption of PEG-40 HCO on the carbon steel surface in SFGE follows Langmuir adsorption isotherm.^25^

The value of adsorption equilibrium constant *K*_ads_ is calculated from the reciprocal of the intercept of the isotherm line as 4.6 × 10^4^ M^−1^. *K*_ads_ that is related to the standard free energy of adsorption (Δ*G*^0^_ads_) by:^26,27^

4 Δ*G*^0^_ads_ = −*RT* ln(55.5 *K*_ads_)

where *R* is the molar gas constant, *T* is the absolute temperature and 55.5 is the concentration of water in solution expressed in molar.

The values of the free energy of adsorption Δ*G*^0^_ads_ as calculated from the Langmuir adsorption isotherm was −36.5 kJ mol^−1^. The negative value of Δ*G*^0^_ads_ ensures the spontaneity of the adsorption of PEG-40 HCO on the carbon steel surface in SFGE.^28^

### 3.4. Activation energy and heat of adsorption

The effect of increase in solution temperature from 298 to 323 K on *j*_corr_ and *θ* is summarized in Table 3.

**Table 3:** The values of corrosion current densities (*j*_corr_), degrees of surface coverage (*θ*), activation energy (*E*_a_) and heat of adsorption (*Q*_ads_) for carbon steel in SFGE in the absence and presence of different concentration of PEG-40 HCO at 298 and 323 K

PEG-40 HCO ppm	*j*_corr1_ (nA cm^−2^), 298 K	*j*_corr2_ (nA cm^−2^), 323 K	*θ*_1_, 298 K	*θ*_2_, 323 K	*E*_a_, kJ mol^−1^	*Q*_ads_, kJ mol^−1^
Blank	630	985	—	—	14.29	—
20	263	739	0.582	0.250	33.04	−45.35
40	190	653	0.698	0.337	39.48	−48.17
60	141	559	0.775	0.432	44.04	−48.26
80	96	465	0.847	0.528	50.45	−51.13
100	39	376	0.938	0.618	72.46	−71.44

The apparent activation energy *E*_a_ of the corrosion reaction was calculated using the Arrhenius equation:^29^

5 log(*j*_corr2_/*j*_corr1_) = *E*_a_/2.303*R*[(1/*T*_1_) − (1/*T*_2_)]

where *j*_corr1_ and *j*_corr2_ are the corrosion current densities at temperature *T*_1_ and *T*_2_, respectively.

An estimate of heat of adsorption *Q*_ads_ was obtained from the trend of surface coverage *θ* with temperature as follows:^30^

6 *Q*_ads_ = 2.303*R*[log(*θ*_2_/1 − *θ*_2_) − log(*θ*_1_/1 − *θ*_1_)] × (*T*_1_*T*_2_/*T*_2_ − *T*_1_)

where *θ*_1_ and *θ*_2_ are the degrees of surface coverage at temperatures *T*_1_ and *T*_2_. The calculated values for *E*_a_ and *Q*_ads_ are given in Table 3.

As it can be seen from Table 3, that the rates of carbon steel corrosion in presence of steel in the simulated fuel-grade ethanol increased with temperature while the inhibition efficiency decreased.

From the Table 3, it is clear that the lower values of *E*_a_ obtained in presence of PEG-40 HCO compared with those obtained in its absence can be attributed to its physical adsorption on the metal surface.^31^ Additionally, since *Q*_ads_ have negative values, it can be concluded that the degree of surface coverage by PEG-40 HCO molecule decreased with rise in temperature.^31^ This can be interpreted on the basis that increasing temperature leads to the desorption of some adsorbed PEG-40 HCO molecule from the carbon steel surface.

### 3.5. Quantum chemical calculations

**Fig. 7:**
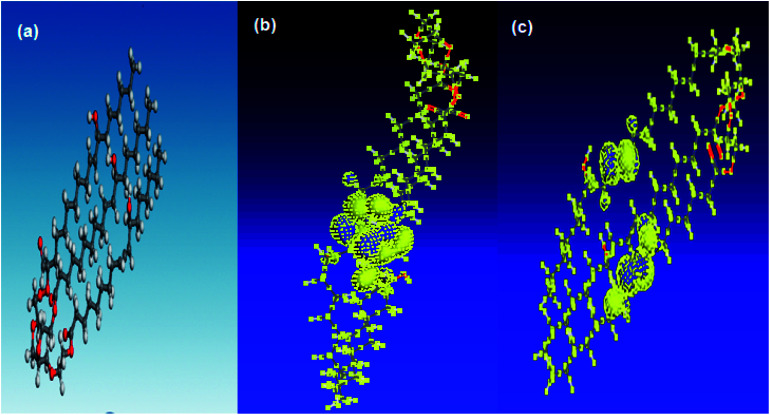
(a) The optimized geometry, (b) HOMO, (c) LUMO density distribution for PEG-40 HCO.

Fig. 7 shows the optimized geometry, HOMO, LUMO density distribution for PEG-40 HCO. The zones of the HOMO are the sites liable to electrophilic attack and represent the active centers for the adsorption process. LUMO zones (anti-bonding orbital) can receive electrons from Fe d-orbital.^32^ The high value of *E*_HOMO_ (*E*_HOMO_ = −9.024 eV) refers to the ability of the PEG-40 HCO molecule to give electrons to empty d-orbitals of Fe atom to form coordinate bond.^33^ On other hand, the low value of *E*_LUMO_ (*E*_LUMO_ = −0.644 eV) confirm the ability of the PEG-40 HCO molecule to accept electrons from filled Fe d-orbital. Based on the above data, it clears that The *E*_HOMO_ and *E*_LUMO_ are related to the degree of adsorption of PEG-40 HCO molecule on the Fe surface. Where the low value of Δ*E* (Δ*E* = 8.38 eV) provides good inhibition efficiency.^34^ These theoretical calculations agree well with the data obtained experimentally.

## 4. Conclusions

(1) Electrochemical measurements (cyclic voltammetry and potentiodynamic polarization) were devoted to test the corrosion behavior of carbon steel in the simulated fuel-grade ethanol SFGE and the inhibition characters of PEG-40 HCO.

(2) Voltammetry shows that the anodic current density value enhances and *E*_pit_ move in the active direction with increasing water and chloride ions concentration.

(3) SEM images confirmed the existence of pits on the carbon steel surface exposed to SFGE, as the water content increases from 1.0 to 3.0 vol%.

(4) The results obtained form Tafel curves indicate that PEG-40 HCO has good inhibition efficiency for carbon steel in SFGE.

(5) The inhibition effect of PEG-40 HCO is due to its physical adsorption on the carbon steel surface. The adsorption follows Langmuir adsorption isotherm.

(6) The degree of surface coverage by PEG-40 HCO decreased with temperature.

(7) Both experimental and quantum chemical calculations are in excellent agreement.

## Conflicts of interest

There are no conflicts to declare.

## Supplementary Material

## References

[cit1] Cardona C. A., Sánchez Ó. J. (2007). Bioresour. Technol..

[cit2] Sridhar N., Price K., Buckingham J., Dante J. (2006). Corrosion.

[cit3] Maldonado J. G. and Sridhar N., SCC of carbon steel in fuel ethanol service, in: Effect of Corrosion Potential and Ethanol Processing Source, Corrosion 2007, NACE International, 2007, Nashville, TN, Paper No. 07574

[cit4] Lou X., Yang D., Singh P. (2009). Corrosion.

[cit5] Lou X., Singh P. M. (2010). Corros. Sci..

[cit6] Heitz E. (1974). Adv. Corros. Sci. Technol..

[cit7] Avelar H. D. M., Barbeira P. J. S. (2007). Fuel.

[cit8] Deyab M. A. (2016). J. Taiwan Inst. Chem. Eng..

[cit9] Deyab M. A. M. (2015). J. Surfactants Deterg..

[cit10] Deyab M. A., El Bali B., Essehli R., Ouarsal R., Lachkar M., Fuess H. (2016). J. Mol. Liq..

[cit11] ASTM International , ASTM 4806, West Conshohocken, PA, USA, 2010

[cit12] Banas J., Stypula B., Banas K., Swiatowska-Mrowiecka J., Starowicz M., Lelek-Borkowska U. (2009). J. Solid State Electrochem..

[cit13] Deyab M. A. (2016). Electrochim. Acta.

[cit14] Deyab M. A. (2009). J. Solid State Electrochem..

[cit15] Banas J. (1987). Electrochim. Acta.

[cit16] Brossia C. S., Gileadi E., Kelly R. G. (1995). Corros. Sci..

[cit17] Szklarska-Smialowska Z. , Pitting corrosion of metals, NACE, Houston, 1986

[cit18] Deyab M. A. (2016). Desalination.

[cit19] Deyab M. A., Essehli R., El Bali B. (2015). RSC Adv..

[cit20] John S., Joseph A., Sajini T., Jose A. J. (2017). Egypt. J. Pet..

[cit21] Deyab M. A. (2016). RSC Adv..

[cit22] Deyab M. A., Abo Dief H. A., Eissa E. A., Taman A. R. (2007). Electrochim. Acta.

[cit23] Yüce A. O., Kardaş G. (2012). Corros. Sci..

[cit24] Deyab M. A., Ouarsal R., Lachkar M., El Bali B., Essehli R. (2016). J. Mol. Liq..

[cit25] Oguzie E. E., Njoku V. O., Enenebeaku C. K., Akalezi C. O., Obi C. (2008). Corros. Sci..

[cit26] Deyab M. A., Essehli R., El Bali B. (2015). RSC Adv..

[cit27] Umoren S. A., Obot I. B., Ebenso E. E., Okafor P. C., Ogbobe O., Oguzie E. E. (2006). Anti-Corros. Methods Mater..

[cit28] Oguzie E. E. (2008). Port. Electrochim. Acta.

[cit29] Deyab M. A. (2016). J. Taiwan Inst. Chem. Eng..

[cit30] Deyab M. A., Eddahaoui K., Essehli R., Rhadfi T., Benmokhtar S., Mele G. (2016). Desalination.

[cit31] Oguzie E. E. (2004). Mater. Chem. Phys..

[cit32] Sastri V. S., Perumareddi J. R. (1997). Corrosion.

[cit33] Martinez S. (2003). Mater. Chem. Phys..

[cit34] Bahrami M. J., Hosseini S. M. A., Pilvar P. (2010). Corros. Sci..

